# Supplementary material for: Genome-Wide Identification and Characterization of Argonaute, Dicer-like and RNA-Dependent RNA Polymerase Gene Families and Their Expression Analyses in Fragaria spp
Source: Genes (Basel). 2023 Jan 1;14(1):121. doi: 10.3390/genes14010121 (PMC9859564; doi:10.3390/genes14010121)
Supplement: Supplementary file 1 [file genes-14-00121-s001.zip › genes-2078922-supplementary.pdf]

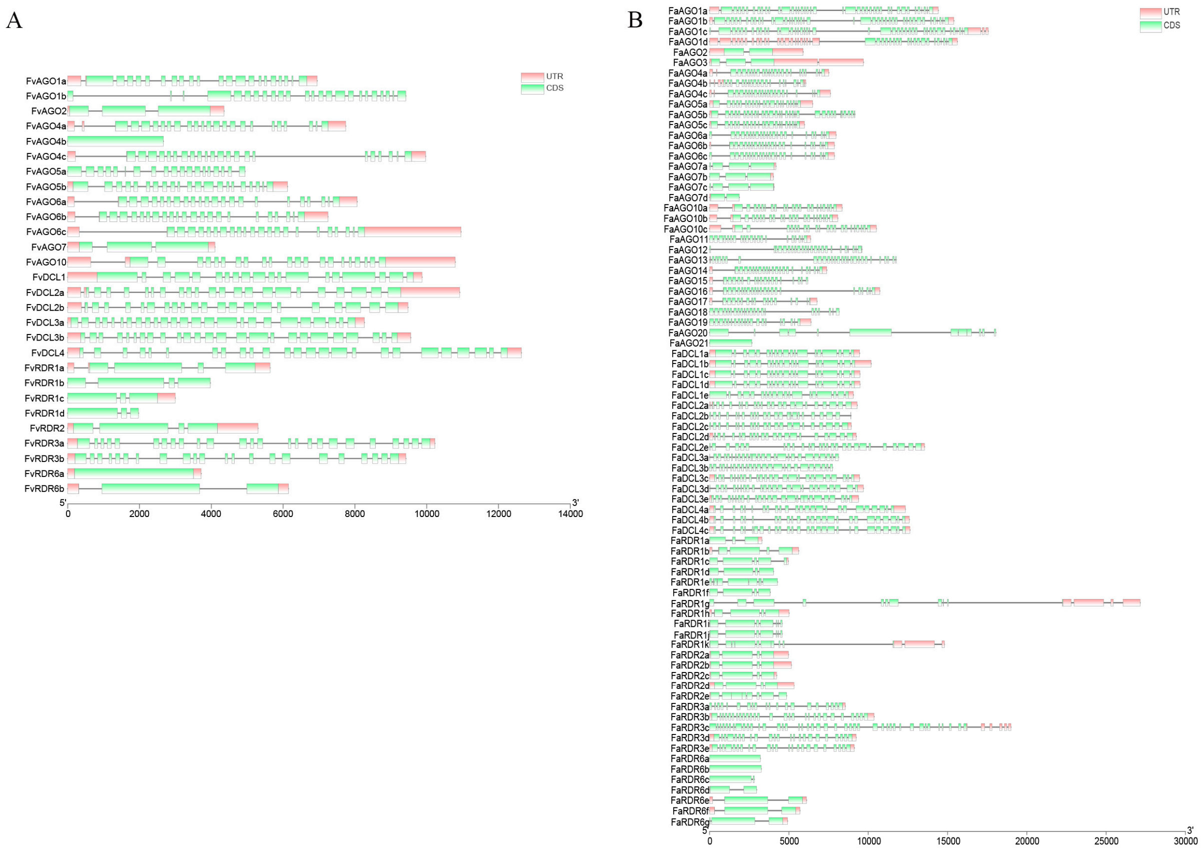

Figure S1: The gene structures of AGO, DCL, and RDR genes in F. vesca (**A**) and F. ×ananassa (**B**).

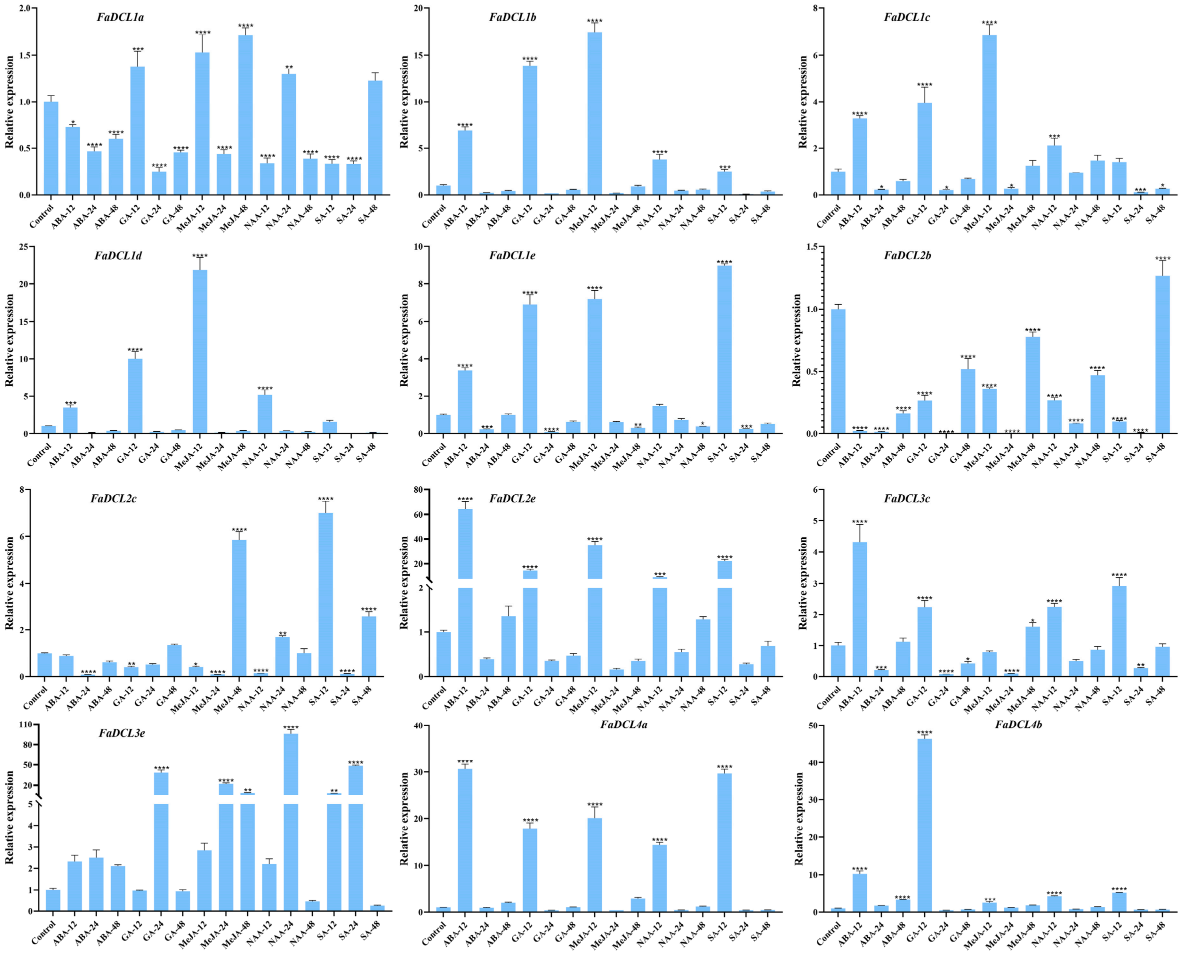

Figure S2: qRT-PCR analyses of FaDCL genes expression under five hormone treatments. All the treatments are compared with "Control", and * represents $p \leq 0.05$, ** represents $p \leq 0.01$, *** represents $p \leq 0.001$, **** represents $p \leq 0.0001$.

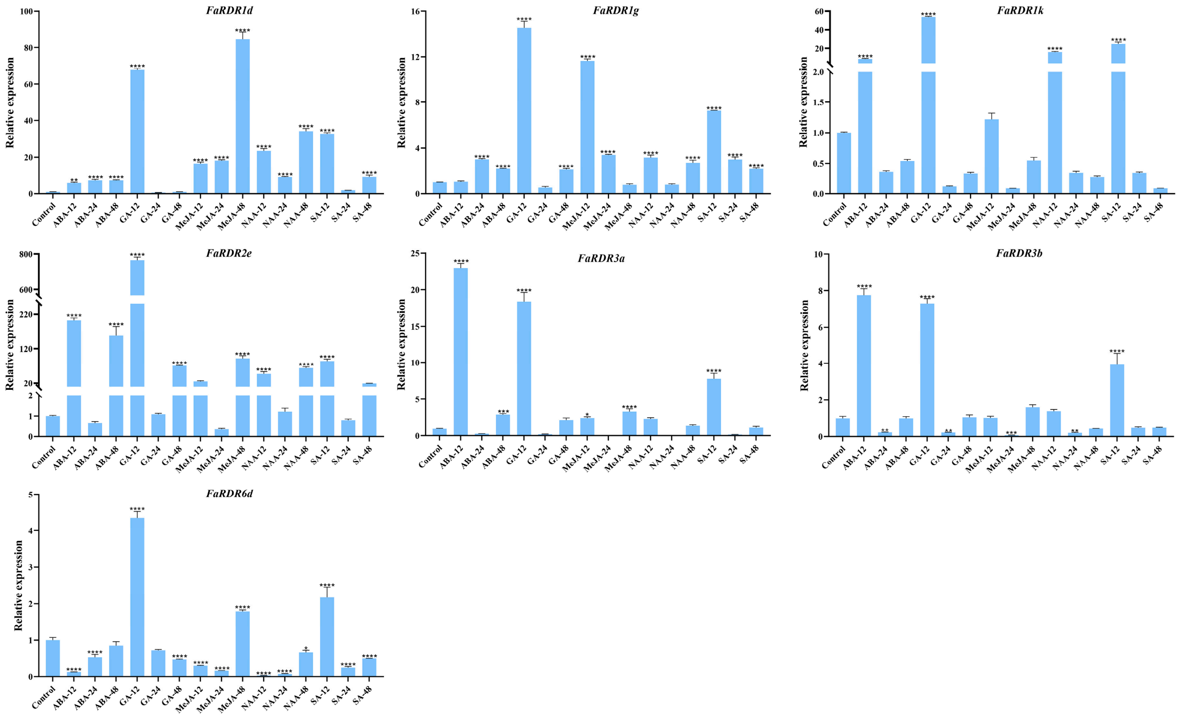

Figure S3: Expression profiles of strawberry RDRs analyzed by qRT-PCR after five hormone treatments. All the treatments are compared with "Control", and * represents $p \leq 0.05$, ** represents $p \leq 0.01$, *** represents $p \leq 0.001$, **** represents $p \leq 0.0001$.
